# *DiffLogo*: a comparative visualization of sequence motifs

**DOI:** 10.1186/s12859-015-0767-x

**Published:** 2015-11-17

**Authors:** Martin Nettling, Hendrik Treutler, Jan Grau, Jens Keilwagen, Stefan Posch, Ivo Grosse

**Affiliations:** 1 Institute of Computer Science, Martin Luther University Halle-Wittenberg, Halle (Saale), Germany; 2 Leibniz Institute of Plant Biochemistry, Halle (Saale), Germany; 3 Institute for Biosafety in Plant Biotechnology, Julius Kühn-Institut (JKI), Federal Research Centre for Cultivated Plants, Quedlinburg, Germany; 4 German Centre for Integrative Biodiversity Research (iDiv) Halle-Jena-Leipzig, Leipzig, Germany

**Keywords:** Sequence analysis, Sequence logo, Sequence motif, Position weight matrix, Binding sites

## Abstract

**Background:**

For three decades, sequence logos are the *de facto* standard for the visualization of sequence motifs in biology and bioinformatics. Reasons for this success story are their simplicity and clarity. The number of inferred and published motifs grows with the number of data sets and motif extraction algorithms. Hence, it becomes more and more important to perceive differences between motifs. However, motif differences are hard to detect from individual sequence logos in case of multiple motifs for one transcription factor, highly similar binding motifs of different transcription factors, or multiple motifs for one protein domain.

**Results:**

Here, we present *DiffLogo*, a freely available, extensible, and user-friendly R package for visualizing motif differences. *DiffLogo* is capable of showing differences between DNA motifs as well as protein motifs in a pair-wise manner resulting in publication-ready figures. In case of more than two motifs, *DiffLogo* is capable of visualizing pair-wise differences in a tabular form. Here, the motifs are ordered by similarity, and the difference logos are colored for clarity. We demonstrate the benefit of *DiffLogo* on CTCF motifs from different human cell lines, on E-box motifs of three basic helix-loop-helix transcription factors as examples for comparison of DNA motifs, and on F-box domains from three different families as example for comparison of protein motifs.

**Conclusions:**

*DiffLogo* provides an intuitive visualization of motif differences. It enables the illustration and investigation of differences between highly similar motifs such as binding patterns of transcription factors for different cell types, treatments, and algorithmic approaches.

**Electronic supplementary material:**

The online version of this article (doi:10.1186/s12859-015-0767-x) contains supplementary material, which is available to authorized users.

## Background

Biological polymer sequences encode information by the order of their monomers, i.e., bases or amino acids. Often specific parts of the polymer sequence are of particular interest, as they encode, for instance, the binding of transcription factors to specific binding sites [1, 2], the binding to micro-RNA-targets in mRNAs, splice donor sites and splice acceptor sites in pre-mRNAs [3, 4], the presence of phosphorylation sites in proteins, or the folding of specific protein domains [5]. The set of subsequences of one specific biological process are often represented as a sequence motif.

A sequence motif is a model, that represents the preference for the monomers based on a set of aligned biopolymer sequences. Sequence motifs are the result of pipelines comprising wet-lab experiments and motif prediction algorithms, and are frequently used as the basis of *in silico* predictions [6]. Thus, sequence motif are critical for research of a wide range of problems in biology and bioinformatics.

Considering a particular transcription factor, there are many pipelines that combine wet-lab experiments such as *HT-SELEX* [7, 8], *ChIP-Seq* [9] or *DNase-Seq footprinting* [10] with motif prediction algorithms such as *MEME* [2, 11], *ChIPMunk* [12], *POSMO* [13], or *Dimont* [14]. Wet-lab experiments differ in their experimental setup, e.g., ecotypes, cell types, developmental stage, time points, or treatment, and motif prediction algorithms differ in their mathematical theory and implementation details.

Visualizing the results of motif discovery is nowadays accomplished by sequence logos [15], the *de facto* standard for visualizing motifs in biology and bioinformatics. Sequence logos emerged as an essential tool for researchers to interpret findings, document work, share knowledge, and present results.

**Fig. 1:**
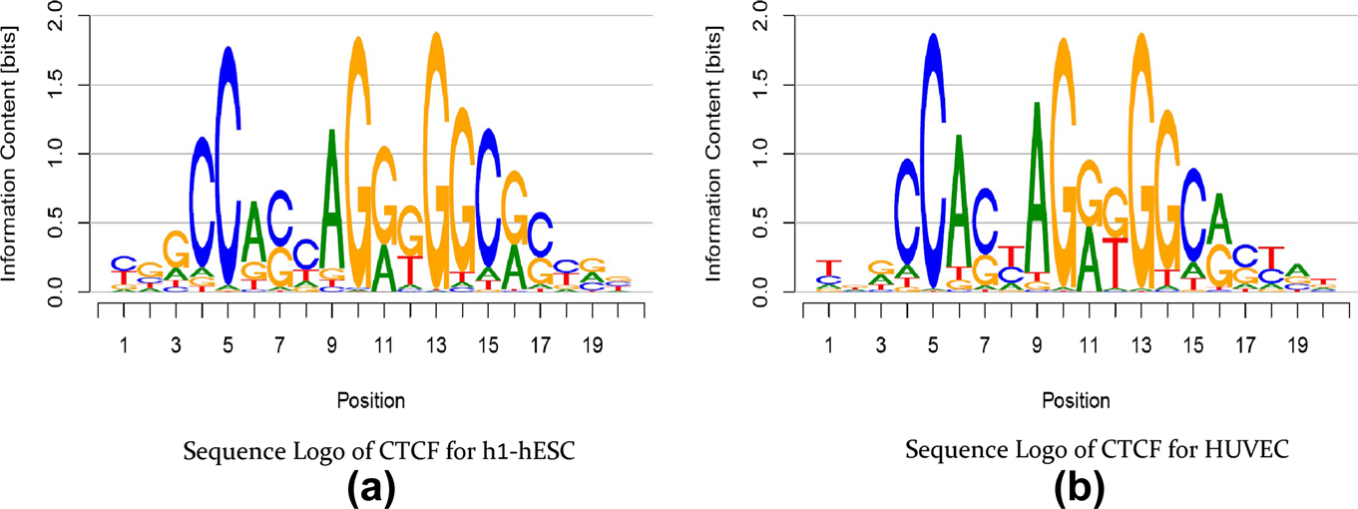
Sequence logos of CTCF motifs from cell lines H1-hESC and HUVEC. The two sequence logos are highly similar in their conservation profile (height of stacks) and nucleotide preference at the individual motif positions

However, comparing multiple sequence logos by visual inspection is sometimes tricky. Differences between sequence logos of two unrelated transcription factors are usually obvious, whereas differences between sequence logos of the same transcription factor are often less obvious and rather hard to perceive as depicted in Fig. 1. Moreover, the results of motif discovery algorithms need to be compared against huge reference databases such as *JASPAR* [16] or *UniProbe* [17] or motifs from literature.

For this reason, the comparison of motifs is of primary interest. Several numerical measures including variants of Euclidean distance, Pearson correlation, and Jensen-Shannon divergence have been used to compare motifs [18–21]. These measures express the difference of motifs as a single number that can be easily utilized subsequently, e.g., for rankings or clustering algorithms. However, these measures lose the information of what exactly makes the difference between the motifs of interest. Hence, the comparison of multiple pairs of motifs can result in similar measures.

There are various tools for the analysis and visualization of motifs as summarized in Table 1. The R package *seqLogo* [22] is an implementation of sequence logos. In the context of motif comparison, sequence logos may be interpreted as a comparison of the input motif with a uniformly distributed motif. The web application *iceLogo* [23] extends this approach by comparing the input motif with a motif that follows the same background distribution at each motif position. Basically, *seqLogo* and *iceLogo* are designed for the presentation of single motifs. In contrast, the R package *MotifStack* [24] and the web application *STAMP* [25] are designed for the presentation of multiple motifs. Here, the input motifs are clustered and presented as sequence logos. Thus, the approach of both tools may be interpreted as multiple comparisons with a uniformly distributed motif. The web application *Two Sample Logo* [26] is capable of comparing two input motifs on the basis of probability theory. This comparison is performed for each motif position individually and results in a sophisticated motif comparison. Depending on the focus of each tool, the input format is a set of aligned sequences and/or a position frequency matrix or position weight matrix. In addition, some tools focus exclusively on DNA motifs, while others cover DNA, RNA, and protein motifs or even allow arbitrary alphabets. Table 1 summarizes tools and their capabilities. In section 4 of Additional file 1, we additionally provide comparative example plots generated by *seqLogo*, *iceLogo*, *STAMP*, *Two Sample Logo*, and *DiffLogo*.

**Table 1:** Comparison of related tools. We compare six publicly available tools on the basis of five criteria

	Features				
Tools	Alphabet	Input format	Comparison	Clustering	Extensible
*seqLogo*	DNA	matrix	uniform	-	-
*iceLogo*	DNA/RNA, proteins	sequences	average	-	-
*MotifStack*	any	matrix	uniform	hclust	-
*STAMP*	DNA	sequences, matrix	uniform	UPGMA/SOTA	-
*Two Sample Logo*	DNA/RNA, proteins	sequences	position-specific	-	-
*DiffLogo*	any	sequences, matrix	position-specific	hclust, optimal leaf ordering	✓

In the first and second column, we examine the kind of supported input, in the third and forth column we examine the mode of action, and in the fifth column we examine whether the tool is extensible. For the criterion “alphabets” we summarize the supported biopolymers out of DNA, RNA, and proteins or arbitrary alphabets in case of “any”. For the criterion “input format” we discriminate a set of “sequences” versus “matrix”, which addresses at least one out of the formats position weight matrix (PWM), position frequency matrix (PFM), and position count matrix (PCM). For the criterion “comparison” we characterize the kind of distribution that is used for motif comparison (“uniform” is the uniform distribution, “average” is the average base distribution in a set of sequences, and “position-specific” is a position-specific distribution). For the criterion “clustering” we point out whether there is a clustering of motifs and which cluster-algorithm is used. For the criterion “extensible” we note whether the tool is extensible by the user

We intend the pair-wise comparison of motifs and extend this idea towards the comparison of multiple motifs as follows.

We focus on the comparison of position-specific symbol distributions of two motifs. We neglect dependencies between different motif positions to reduce complexity. As suggested by the *sequence logo* approach, we intend to represent the characteristics of each motif position by the two properties stack height and symbol height within a stack. The stack height is to be proportional to the degree of distribution dissimilarity. The symbol height is to be proportional to the degree of differential symbol abundance.

We intend to compare three or more motifs on the basis of pair-wise motif comparisons. This comparison is to take into account all pair-wise motif comparisons, suggesting an arrangement in a grid with one row and one column for each motif and one cell for each motif comparison. Similar motifs are to be placed in nearby rows and columns, and the degree of similarity between all motifs is to become obvious at a glance analogous to heatmaps. The grid is to be complemented with a display of the individual sequence logos for further comparisons.

## Implementation

In this section, we first define the used notation. We then briefly describe the classical sequence logo. Subsequently, we introduce the difference logo for the visualization of pair-wise motif differences. We discuss this new method and explore potential biological interpretations. Finally, we propose an approach for employing difference logos for the joint comparison of multiple motifs.

### Basic notation and sequence logo

Consider a motif as an abstract description of a given set of aligned sequences of common length *L* from the alphabet $\mathcal {A}$. The relative frequency of symbol $a \in \mathcal {A}$ at position *ℓ*∈ [ 1,*L*] corresponds to the (estimated) probability *p*_*ℓ*,*a*_. In case of two motifs, we use *p*_*ℓ*,*a*_ for the first motif and analogously *q*_*ℓ*,*a*_ for the second motif.

The well-known sequence logo visualizes a motif with a symbol stack for each position. We denote the height of the stack at position *ℓ* by *H*_*ℓ*_ and the height of symbol *a* within this stack by *H*_*ℓ*,*a*_. In the traditional sequence logo, *H*_*ℓ*_ and *H*_*ℓ*,*a*_ are defined by

(1) $$\begin{array}{@{}rcl@{}} H_{\ell} &=& \log_{2}(|\mathcal{A}|) - \sum_{a \in \mathcal{A}} p_{\ell,a} \cdot \log_{2}(p_{\ell,a}) \end{array} $$

(2) $$\begin{array}{@{}rcl@{}} H_{\ell,a} &=& p_{\ell,a} \cdot H_{\ell}, \end{array} $$

which states that the height of a stack at position *ℓ* reflects the degree of conservation at position *ℓ* quantified by the information content and that the height of each symbol at position *ℓ* is proportional to its frequency at position *ℓ*. Hence, the traditional sequence logo is an intuitive visualization of both (i) conserved motif positions and (ii) abundant bases.

### The approach of *DiffLogo*

As specified earlier, we compare motifs per position. Similar to the sequence logo, we show a symbol stack for each position. We redefine the calculation of *H*_*ℓ*_ and use this measure as the total height of position *ℓ* reflecting the difference of the symbol distribution of both motifs at this position. We redefine the calculation of *H*_*ℓ*,*a*_ and use this measure as the height of a symbol within the stack at position *ℓ*. In the following, *H*_*ℓ*,*a*_ can be positive or negative. Symbols with positive values *H*_*ℓ*,*a*_ are plotted upward. Symbols with negative values *H*_*ℓ*,*a*_ are plotted downward.

Generally, there is a plethora of well-understood mathematical criteria that can be combined to define the height of a symbol stack and the relative heights of symbols within the stack such as probability differences, information divergences, distance measures, or entropies [27]. In the following, we present *DiffLogo* with the example of the Jensen-Shannon divergence for the calculation of *H*_*ℓ*_ and normalized probability differences for the calculation of *H*_*ℓ*,*a*_. We denote the combination of these two measures as weighted difference of probabilities.

### Weighted difference of probabilities

**Fig. 2:**
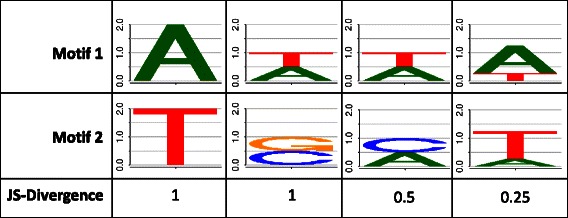
Exemplary comparison of four DNA motifs of length one using the Jensen-Shannon divergence. Motif 1 and motif 2 are depicted as sequence logos. For each column, we compare the motif in the first row with the motif in the second row using the Jensen-Shannon divergence listed in the third row. In the first example we depict the case with only one base in each motif resulting in a maximal Jensen-Shannon divergence of 1 bit. In the second example we depict the case with two equally abundant bases both in motif 1 and motif 2 (both different) resulting again in a maximal Jensen-Shannon divergence of 1 bit. In the third example we depict the case with two equally abundant bases both in motif 1 and motif 2 (one equal and one different) resulting in a Jensen-Shannon divergence of 0.5 bit. In the fourth example we depict the case with two bases both in motif 1 and motif 2 (differentially abundant) resulting in a Jensen-Shannon divergence of 0.25 bit

We calculate the stack height for each motif position using the Jensen-Shannon divergence. The Jensen-Shannon divergence is a measure for the dissimilarity of two probability distributions based on information theory [28] (see Fig. 2). In contrast to other measures, the Jensen-Shannon divergence shows a comparable behavior when evaluating dissimilarities of distributions near the uniform distribution. The Jensen-Shannon divergence of two motifs at position *ℓ* is given by

(3) $$\begin{array}{*{20}l} H_{\ell} = \frac{1}{2} \sum_{a \in \mathcal{A}} p_{\ell,a} \log_{2} \frac{p_{\ell,a}} {m_{\ell,a}}+ \frac{1}{2} \sum_{a \in \mathcal{A}} q_{\ell,a} \log_{2} \frac{q_{\ell,a}}{m_{\ell,a}}, \end{array} $$

where $m_{\ell,a} = \frac {p_{\ell,a} + q_{\ell,a}}{2}$.

We define the height of each symbol by

(4) $$\begin{array}{*{20}l} H_{\ell,a} &= r_{\ell,a} \cdot H_{\ell}, \end{array} $$

where we define the weight *r*_*ℓ*,*a*_ as

(5) $$\begin{array}{*{20}l} r_{\ell,a} &=\left\{ \begin{array}{ll} \frac{p_{\ell,a} - q_{\ell,a}}{\sum_{a' \in \mathcal{A}} |p_{\ell,a'} - q_{\ell,a'}|} & \text{if } p_{\ell} \neq q_{\ell}\\ 0 & \text{otherwise.} \end{array} \right. \end{array} $$

*r*_*ℓ*,*a*_ is the probability difference of symbol *a* at position *ℓ* between two motifs normalized by the sum of absolute probability differences at this position. We use normalized probability differences as these are indicators for the gain or loss of symbol abundance and provide a view on the symbol distribution differences of both motifs. As a consequence, symbols less abundant in the second motif compared to the first motif are plotted upward, and symbols more abundant in the second motif compared to the first motif are plotted downward.

This representation emphasizes a high gain or loss of probability in co–occurrence with a high gain or loss of information content. The sum of the heights of symbols with a gain of probability and the sum of the heights of symbols with a loss of probability are equal at every position, because each gain of probability of one symbol implies a loss of probability of the remaining symbols. The advantage of this approach is that we are capable of seeing differences of position-specific symbol distributions and of seeing those symbols that are responsible for these differences by gaining or losing abundance.

### Comparison of multiple motifs

According to the requirements formulated above, we propose a visualization for the joint comparison of *N*≥3 motifs given the measure *H*_*ℓ*_ as follows.

We plot the difference logos of all *N*×(*N*−1) motif pairs with a common ordinate scaling. We define a scalar dissimilarity value *D* for a pair of motifs as the sum of all stack heights in the corresponding difference logos,

(6) $$\begin{array}{*{20}l} D &=\sum_{\ell = 1}^{L} H_{\ell}. \end{array} $$

We compute a motif order to group similar motifs. Here, we take the optimal leaf order of a hierarchical clustering of the motifs based on *D* (function *hclust* in R package *stats* and function *order.optimal* in R package *cba*). We arrange the difference logos ordered in an *N*×*N* grid with an empty diagonal. Difference logos opposing each other across the diagonal of the grid correspond to each other by an inversion of the ordinate. We visualize *D* with the background color of the corresponding difference logo using a color gradient from green (most similar among all pairwise comparisons) to red (most dissimilar). We outline the motif names above each column and left of each row. In addition, we allow the possibility of drawing the classic sequence logos and the cluster tree above the columns as auxiliary information.

The advantage of this approach is that we are capable of surveying the overall similarities and dissimilarities in the resulting difference logo grid. Greenish regions indicate similar motif groups and reddish rows and columns indicate less similar motifs. Given a region of interest, it is furthermore possible to comprehend the origins of dissimilarities from the individual difference logos and optionally the sequence logos.

### R package

*DiffLogo* is written in R [29]. We provide the implementation as a ready-to-use R package. For symbol drawing, *DiffLogo* uses adapted methods from the package *seqLogo* [22] in the software suite *bioconductor* [30]. *DiffLogo* allows the analysis of sequence motifs defined over arbitrary alphabets.

The core functions can be parameterized with functions for *H*_*ℓ*_ and *r*_*ℓ*,*a*_. Hence, the user is capable of combining different formulae for *H*_*ℓ*_ and *r*_*ℓ*,*a*_. We provide implementations of the Jensen-Shannon divergence and the normalized probability difference used for the difference logos presented in this manuscript. In addition, *DiffLogo* provides other implementations for *H*_*ℓ*_ and *r*_*ℓ*,*a*_ as alternatives. Exemplarily, we show the result of eight different combinations of measures for stack height and symbol height in Additional file 1: Tables S1 and S2. The *DiffLogo* package comprises example data, example code, and further documentation.

## Results and discussion

In this section, we present three examples demonstrating the utility of *DiffLogo* in different applications. First, we examine differences in motifs of DNA binding sites of the same transcription factor from five different cell lines. Second, we examine differences in motifs of DNA binding sites of three different transcription factors with similar binding motifs. Third, we examine differences in motifs of a protein domain.

### DNA motifs of same transcription factor

We consider sequence logos and difference logos of binding sites of the human insulator CTCF in different cell lines as obtained by motif discovery from ChIP-seq data [31] based on preprocessed ChIP-seq data from the ENCODE project. For CTCF motif inference, sequences with *p*-values smaller than 10^-6^ were selected. All data are freely available as Additional File of the original publication [31]. Since CTCF is a DNA-binding protein, the alphabet corresponds to the four nucleotides in this case.

**Fig. 3:**
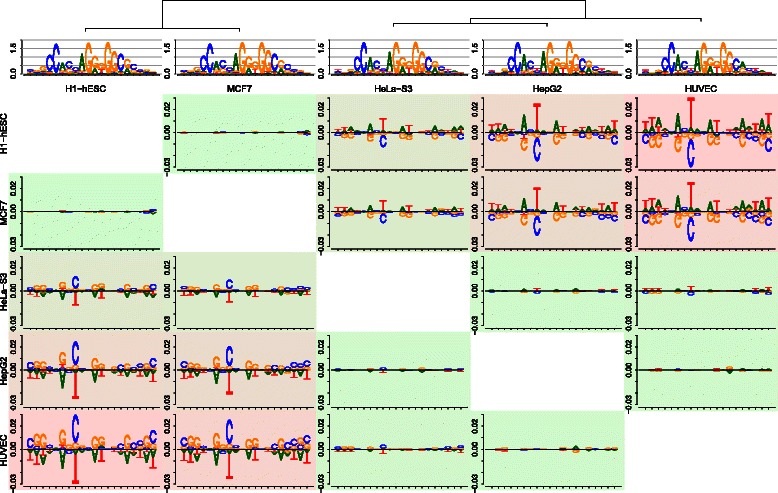
Comparison of five DNA motifs using *DiffLogo*. Comparison of five CTCF motifs from cell lines H1-hESC, MCF7, HeLa-S3, HepG2, and HUVEC. We plot all pair-wise sequence logos and display the distance between each motif using the background color from green (similar) to red (dissimilar). We plot the sequence logos of each motif as well as the leaf-ordered cluster tree above. The motifs of H1-hESC and MCF7 are highly similar and substantially different from the other motifs, while the motifs of HeLe-S3, HepG2, and HUVEC are similar to each other as well. Due to leaf ordering, the difference between compared motifs increases with increasing distance from the main diagonal in the difference logo grid

In Fig. 1, we plot the sequence logos for two of these cell types, namely H1-hESC and HUVEC. Considering the sequence logos, both motifs look highly similar with regard to the conservation as well as the nucleotide preference of individual motif positions, and differences between both motifs are hard to perceive. Considering the corresponding difference logo in Fig. 3 (row 1, column 5 or row 5 column 1), however, we instantly see that indeed a large number of motif positions exhibits differences in nucleotide composition. We find the largest difference according to the difference logo at position 8 of the motifs, where nucleotide C is more prevalent in cell type H1-hESC compared to HUVEC, whereas the opposite holds for nucleotide T. This difference is less visible in the sequence logos, even with hindsight from the difference logo, due to the low conservation at this position. Specifically, the probability of C increases from 0.35 (HUVEC) to 0.58 (H1-hESC), whereas the probability of T drops by a factor of 2 from 0.44 (HUVEC) to 0.21 (H1-hESC). Depending on the application, this difference at position 8 might have a decisive influence on the outcome of, e.g., *in silico* binding site prediction.

In the literature, several positions with substantial motif differences uncovered by *DiffLogo* are known to be related to CTCF binding affinity. For instance [32] show that “low occupancy” CTCF binding sites are enriched for C or G at position 18 compared to “high occupancy” sites, which in our case might indicate that the H1-hESC ChIP-seq data set contains a larger number of such “low occupancy” sites than the HUVEC data set.

In a large-scale study [33], CTCF core motifs are partitioned by the presence or absence of additional upstream and downstream motifs, where the greatest variations in the core motifs between partitions can be found at positions 1-3, 6, 8, 11, 12, 18, and 20, which cover those positions varying in the difference logo. Again, these partitions are related to binding affinity and occupancy of CTCF.

In summary, *DiffLogo* helps to identify several motif positions with substantial variation between cell types, known to be related to CTCF binding affinity and binding site occupancy.

In real-world applications, motifs for more than two cell types are often studied, which might render the pairwise comparison of difference logos a tedious task. We support such an evaluation across multiple cell types by a structured visualization of multiple difference logos as shown in Fig. 3. Here, we compare the pairwise difference logos of CTCF motifs from five cell types, namely H1-hESC, MCF7, HeLa-S3, HepG2, and HUVEC. The cluster tree and background color of the cells are based on numerical measures of motif differences (cf. Implementation) and guide us to the most notable differences between pairs of motifs. For instance, we observe from the tree and background colors that the motifs of H1-hESC and MCF7 are highly similar. The same holds true for the motifs of HeLa-S3, HepG2, and HUVEC, whereas motifs show substantial differences between these two groups. To further facilitate the visual comparison of multiple motifs, we leaf-order the cluster tree such that neighboring motifs are as similar as possible. Due to this ordering, the difference between motif pairs increases with increasing distance from the main diagonal of the difference logo grid. For instance, the topology of the clustering would allow to invert the order of the three leaves under the right sub-tree in Fig. 3, which, however, would bring the quite dissimilar motifs of HUVEC and MCF7 in direct neighborhood. From Fig. 3, we also observe that the two motifs of H1-hESC and HUVEC are the most dissimilar ones among the motifs studied. A visualization of all nine available motifs can be found in Additional file 1: Figure S1.

### DNA motifs of different transcription factors

We demonstrate the utility of *DiffLogo* for motifs derived from binding assays for the human transcription factors Max, Myc, and Mad (Mxi1) from Mordelet *et al*. [34]. These three basic helix-loop-helix transcription factors are members of a regulatory network of transcription factors that controls cell proliferation, differentiation, and cell death. Each transcription factor binds to different sets of target sites, regulates different sets of genes, and thus plays a distinct role in human cells. However, Myc, Max, and Mad have almost identical PWMs, which all correspond to an E-box motif with consensus sequence CACGTG.

The PWMs considered here have been derived from probe sequences and corresponding binding intensities of *in-vitro* genomic context protein-binding microarrays [34]. The exact binding sites within the probe sequences are predicted by the de-novo motif discovery tool Dimont [14] using Slim models [35]. For each of the three transcription factors, the top 1,000 predicted binding sites are used to generate the corresponding PWM.

**Fig. 4:**
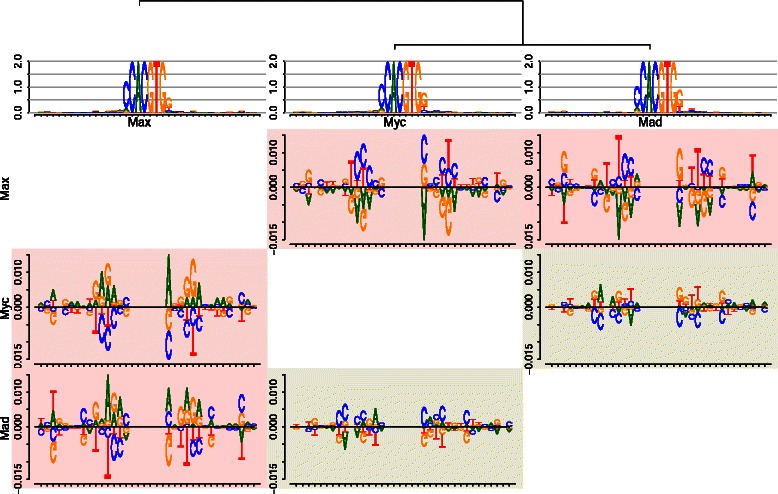
Comparison of E-Box motifs of Max, Myc, and Mad using *DiffLogo*. We plot all pair-wise difference logos and display the distance between each motif using the background color from green (similar) to red (dissimilar). We plot the sequence logos of each motif as well as the leaf-ordered cluster tree above. The motifs of the transcription factors Myc and Mad are more similar to each other than to the motif Max. The six core nucleotides with consensus sequence CACGTG are conserved in the motifs of all three transcription factors and, hence, are not visible in the difference logos

In Fig. 4, we plot the sequence logos and difference logos of Myc, Max, and Mad. We observe from the sequence logos that the binding motifs are almost identical. Considering the difference logos, we observe that the six core nucleotides are conserved in the motifs of all three transcription factors. We find the largest differences between the motif of Max and the motifs of Myc and Mad. In case of Max and Myc, we find a Jensen-Shannon divergence greater than 0.01 bit at positions 11, 12, 22, and 26. In case of Max and Mad, we find a Jensen-Shannon divergence greater than 0.01 bit at positions 3, 12, 22, and 25. In both cases, we mainly find more purine (adenine and guanine) in the motif of Max than in the motifs of Myc and Mad.

### Protein motifs

As a third example, we demonstrate the utility of *DiffLogo* using the F-box domain, which plays a role in protein-protein binding. The complete F-box domain in this example is 48 amino acids long [36]. Here, we investigate the middle section from the 12th to the 35th amino acid.

**Fig. 5:**
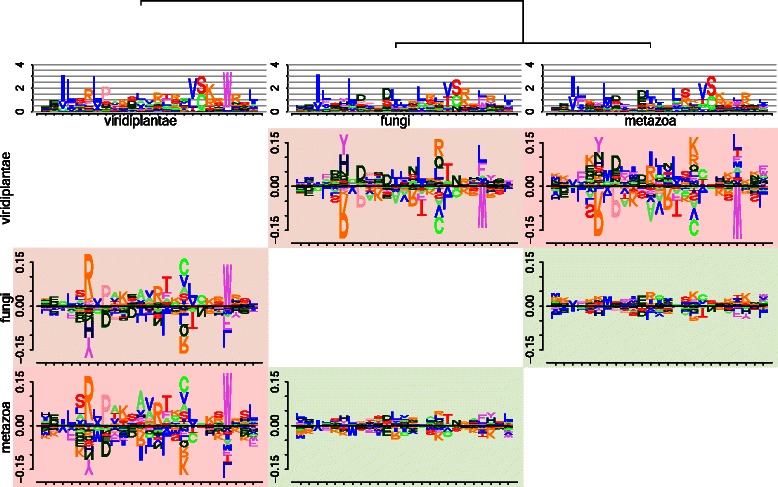
Comparison of three F-box domain motifs using *DiffLogo*. We compare the F-box domains from the kingdoms metazoa, fungi, and viridiplantae and plot all pair-wise difference logos and display the distance between each motif using the background color from green (similar) to red (dissimilar). We plot the sequence logos of each motif as well as the leaf-ordered cluster tree above. The motifs of metazoa and fungi are highly similar. All other pairwise comparisons show substantial differences

In Fig. 5, we plot the sequence logos and difference logos of F-box domains from the three kingdoms metazoa, fungi, and viridiplantae. We observe from the cluster tree and the background colors that the motifs of metazoa and fungi are highly similar, whereas motifs of this group show substantial differences to viridiplantae. The largest difference can be seen between motifs of metazoa and viridiplantae.

When comparing metazoa and fungi with viridiplantae, *DiffLogo* identifies positions 6, 17, and 22 with high values of the Jensen-Shannon divergence. The differences at positions 6 and 22 could be expected from the differences of the sequence logos, whereas the differences at position 17 are not immediately obvious from them. At position 6 the abundance of arginine (R) in viridiplantae is 0.54 and thus more than 10 times higher than in fungi and 12 times higher than in metazoa. At position 22 tryptophane (W) is highly abundant in viridiplantae and 4 and 3.4 times more abundant than in metazoa and fungi. At position 17 the most noticeable differences in viridiplantae to fungi and metazoa can be seen for amino acid cysteine (C), valine (V), alanine (A), and serine (S). The overall abundance increases from 0.13 in metazoa and 0.12 in fungi to 0.64 in viridiplantae. In contrast, the abundance of arginine (R), glutamine (Q), and lysine (K) is only 0.044 in viridiplantae and 0.44 in metazoa and fungi. A visualization of the full F-Box domain from four kingdoms can be found in Additional file 1: Figure S2.

## Conclusion

We present *DiffLogo*, an easy-to-use tool for a fast and efficient comparison of motifs. *DiffLogo* may be applied by users with only basic knowledge in R and is highly configurable and extensible for advanced users. We introduce weighted differences of probabilities to emphasize large differences in position-specific symbol distributions. We present visual comparisons of multiple motifs stemming from motifs of one transcription factor in different cell types, different transcription factors with similar binding motifs, and species-specific protein domains. Figures generated by *DiffLogo* enable the identification of overall motif groups and of sources of dissimilarity. Using *DiffLogo*, it is easily possible to compare motifs from different sources, so *DiffLogo* facilitates decision making, knowledge sharing, and the presentation of results. We make *DiffLogo* freely available in an extensible, ready-to-use R package including examples and documentation. *DiffLogo* is part of *Bioconductor*.

## Availability and requirements

**Project name:** DiffLogo**Project home page:** http://github.com/mgledi/DiffLogo **Availability:** http://bioconductor.org/packages/DiffLogo **Operating system(s):** Platform independent **Programming language:** R **Other requirements:** Installation of R 1.8.0 or higher **License:** LGPL (≥2) **Any restrictions to use by non-academics:** None

## Additional file

Additional file 1
**Supplementary Methods, Results, Figures, and Examples.** This file is structured in four sections. Section 1, *Additional examples*, contains Figures S1 and S2. Figure S1 shows a *DiffLogo* grid for nine CTCF motifs. Figure S2 shows a *DiffLogo* grid for four F-box domain motifs. In section 2, *CTCF with and without clustering*, we show in detail the impact of clustering and optimal leaf ordering for a *DiffLogo* grid of nine CTCF motifs. In section 3, *Alternative combinations of stack heights and symbol weights*, we first describe the mathematical background of four implementations of *H*_*ℓ*_ and two implementations of *r*_*ℓ*,*a*_. Afterwards, we show the result of the eight possible combinations in Tables S1 and S2 on two sequence motifs. In section 4, *Tool comparison*, we compare *DiffLogo* with the five tools *seqLogo*, *iceLogo*, *MotifStack*, *STAMP*, and *Two Sample Logo*. From the set of nine CTCF motifs we selected the pair of motifs with the highest similarity according to the Jensen-Shannon divergence (GM12878 and K562) and the pair of motifs with the lowest similarity according to the Jensen-Shannon divergence (H1-hESC and HUVEC) for the comparison of the five different tools. (PDF 8775 kb)
