# Supplementary material for: DiffLogo: a comparative visualization of sequence motifs
Source: BMC Bioinformatics. 2015 Nov 17;16:387. doi: 10.1186/s12859-015-0767-x (PMC4650857; doi:10.1186/s12859-015-0767-x)
Supplement: Additional file 1 — Supplementary Methods, Results, Figures, and Examples. This file is structured in four sections. Section 1, Additional examples, contains Figures S1 and S2. Figure S1 shows a DiffLogo grid for nine CTCF motifs. Figure S2 shows a DiffLogo grid for four F-box domain motifs. In section 2, CTCF with and without clustering, we show in detail the impact of clustering and optimal leaf ordering for a DiffLogo grid of nine CTCF motifs. In section 3, Alternative combinations of stack heights and symbol weights, we first describe the mathematical background of four implementations of H ℓ and two implementations of r ℓ,a. Afterwards, we show the result of the eight possible combinations in Tables S1 and S2 on two sequence motifs. In section 4, Tool comparison, we compare DiffLogo with the five tools seqLogo, iceLogo, MotifStack, STAMP, and Two Sample Logo. From the set of nine CTCF motifs we selected the pair of motifs with the highest similarity according to the Jensen-Shannon divergence (GM12878 and K562) and the pair of motifs with the lowest similarity according to the Jensen-Shannon divergence (H1-hESC and HUVEC) for the comparison of the five different tools. (PDF 8775 kb) [file 12859_2015_767_MOESM1_ESM.pdf]

1 Additional examples

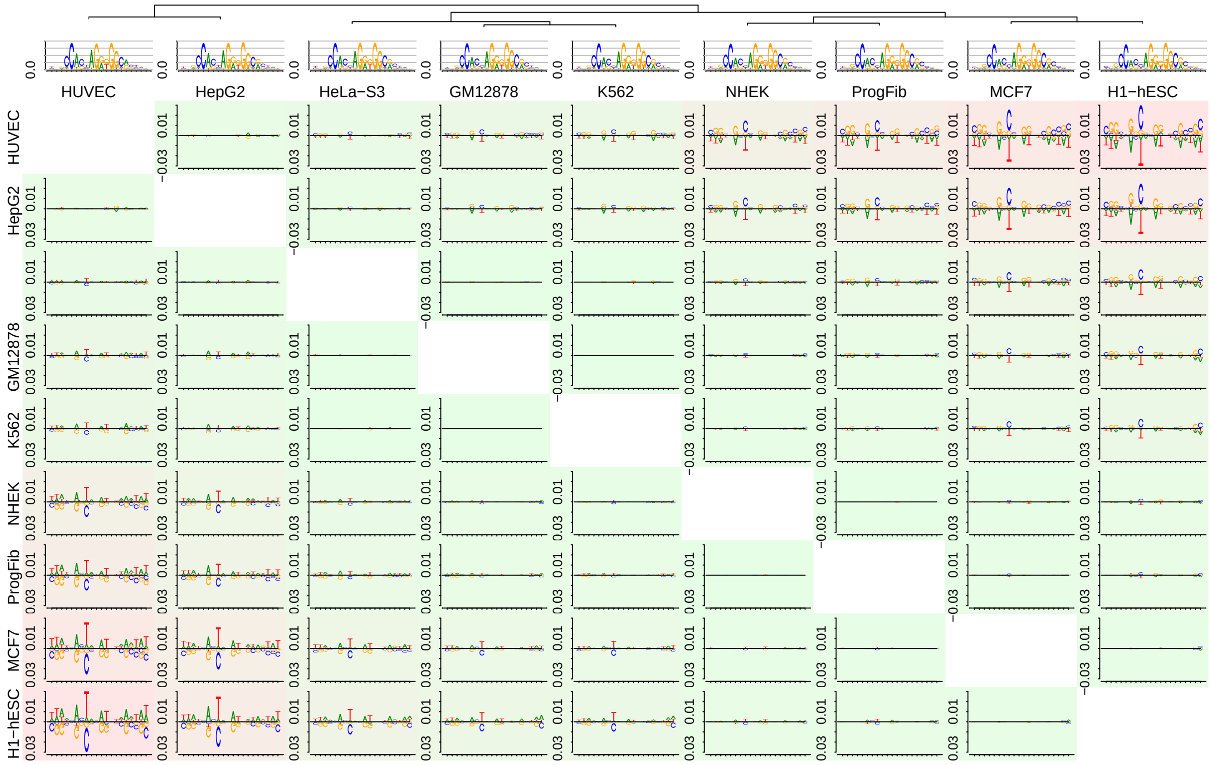

Figure S1: Comparison of nine CTCF motifs from the cell lines HepG2, MCF7, HUVEC, ProgFib, NHEK, K562, HeLa-S3, H1-hESC, GM12878. We plot all pair-wise difference logos and represent the distance between each pair of motifs by the background color from green (similar) to red (dissimilar). We plot the sequence logos of each motif as well as the leaf-ordered cluster tree above.

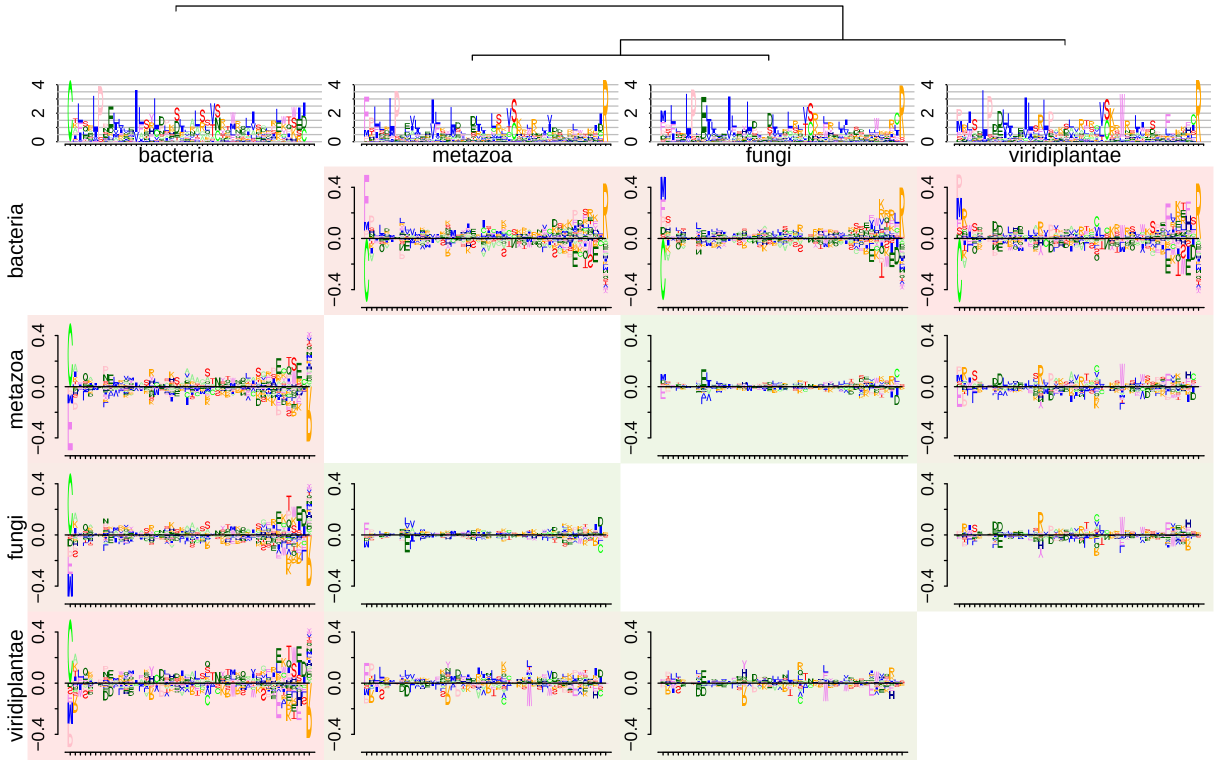

Figure S2: Comparison of F-box domain motifs using *DiffLogo*. Comparison of the F-box domain from the kingdoms bacteria, metazoa, fungi and viridiplantae. We plot all pair-wise difference logos and represent the distance between each pair of motifs by the background color from green (similar) to red (dissimilar). We plot the sequence logos of each motif as well as the leaf-ordered cluster tree above. The motifs of metazoa and fungi are highly similar. Other pair-wise comparisons show substantial differences.

2 CTCF with and without Clustering

The impact of clustering with optimal leaf ordering on the resulting grid of pair-wise comparisons.

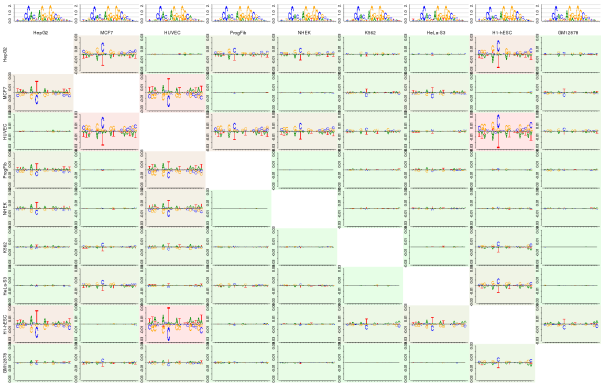

(a) Clustering disabled

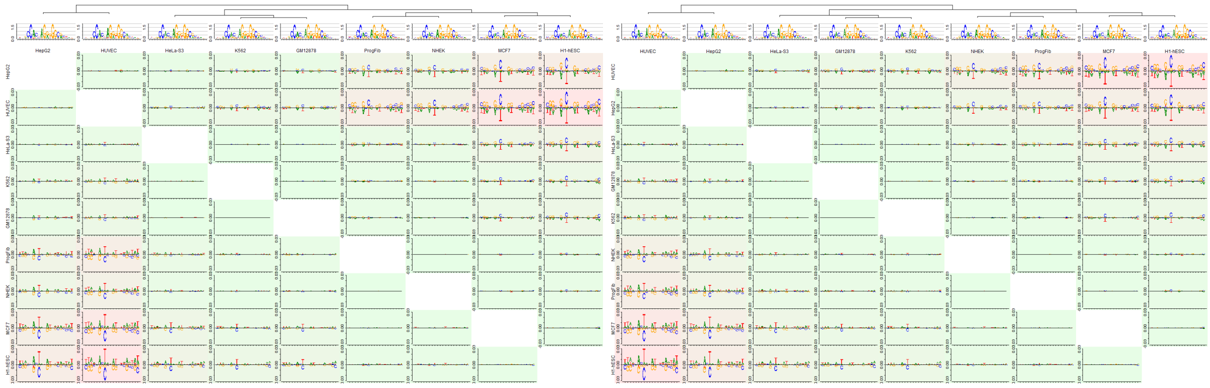

(b) Clustering enabled, optimal leaf ordering disabled (c) Clustering and optimal leaf ordering enabled

Figure S3: **Influence of clustering on appearance of difference logo grid**: a) with clustering disabled, b) with clustering enabled and optimal leaf ordering disabled, and c) with clustering and optimal leaf ordering enabled.

Figure S3 shows the importance of clustering especially when comparing more than four motifs. Without clustering it is hard to recognize groups of similar or dissimilar motifs (Figure S3a). When clustering is enabled, but optimal leaf ordering is disabled, larger groups of similar motifs can be detected. Details are still hard to perceive (Figure S3b). When clustering and optimal leaf ordering are enabled, it is easy to see which two motifs are the most dissimilar ones and it is easy to recognize groups of motifs. Even within these groups it is easy to determine the two motifs that differ the most.

3 Alternative combinations of stack heights and symbol weights

We consider two motifs represented by two PWMs p and q. The height of symbol a in the symbol stack at position ℓ of the difference logo is denoted $H_{\ell,a}$ and given by

$$H_{\ell,a} = r_{\ell,a} \cdot H_\ell,$$

where H_ℓ represents the height of the symbol stack at position ℓ and the weight $r_{\ell,a}$ represents the proportion of symbol $a \in \mathcal{A}$ in the symbol stack at position ℓ, where $\mathcal{A}$ is the alphabet. We calculate $H_{\ell,a}$ for different measures H_ℓ and $r_{\ell,a}$ to emphasize different facets of distribution differences. We propose various alternatives to calculate the measures H_ℓ and $r_{\ell,a}$ as follows (illustrated in supplementary Table S1).

In the following sections, the information content of a PWM p at position ℓ is denoted H_ℓ^p and given by

$$H_\ell^p = \log_2(|\mathcal{A}|) - \sum_{a \in \mathcal{A}} p_{\ell,a} \cdot \log_2(p_{\ell,a}),$$

where $p_{\ell,a}$ is the probability of symbol a at position ℓ in PWM p. H_ℓ^q is defined analogously.

3.1 Different calculations of stack heights H_ℓ

3.1.1 Jensen–Shannon divergence

The Jensen–Shannon divergence is a measure for the difference of two probability distributions based on information theory. The Jensen–Shannon divergence at position ℓ is denoted by $H_\ell^{(\mathrm{i})}$ and given by

$$\begin{aligned} H_\ell^{(\mathrm{i})} =& \frac{1}{2} \sum_{a \in \mathcal{A}} p_{\ell,a} \Big(\log_2(p_{\ell,a}) - \log_2(m_{\ell,a}) \Big) \\ &+ \frac{1}{2} \sum_{a \in \mathcal{A}} q_{\ell,a} \Big(\log_2(q_{\ell,a}) - \log_2(m_{\ell,a}) \Big), \end{aligned}$$

where $m_{\ell,a} = \frac{1}{2}(p_{\ell,a} + q_{\ell,a})$. $H_\ell^{(\mathrm{i})}$ is symmetric and limited to $[0, 1]$. This measure especially emphasizes large distribution differences.

3.1.2 Change of information content (stack)

The change of information content (stack) is a measure for the absolute change of information content between two probability distributions. The change of information content (stack) at position ℓ is denoted by $H_\ell^{(\mathrm{ii})}$ and given by

$$H_\ell^{(\mathrm{ii})} = \sum_{a \in \mathcal{A}} |p_{\ell,a} H_\ell^p - q_{\ell,a} H_\ell^q|.$$

$H_\ell^{(\mathrm{ii})}$ is symmetric and limited to $[0, 2 * \log_2(|\mathcal{A}|)]$. This measure especially emphasizes large changes of information content.

3.1.3 Relative change of information content

The relative change of information content is a measure for the absolute change of information content relative to the average information content of the two probability distributions. The relative change of information content at position ℓ is denoted by $H_\ell^{(\text{iii})}$ and given by

$$H_\ell^{(\text{iii})} = \begin{cases} \frac{\sum_{a \in \mathcal{A}} |p_{\ell,a} H_\ell^p - q_{\ell,a} H_\ell^q|}{\frac{1}{2}(H_\ell^p + H_\ell^q)} & \text{if } p_\ell \neq q_\ell \\ 0 & \text{otherwise.} \end{cases}$$

$H_\ell^{(\text{iii})}$ is symmetric and limited to $[0, 2 * \log_2(|\mathcal{A}|)]$. This measure especially emphasizes large changes of information content relative to the information content of the given distributions.

3.1.4 Change of probabilities (stack)

The change of probabilities (stack) is a measure for the absolute change of probabilities between two probability distributions. The change of probabilities (stack) at position ℓ is denoted by $H_\ell^{(\text{iv})}$ and given by

$$H_\ell^{(\text{iv})} = \sum_{a \in \mathcal{A}} |p_{\ell,a} - q_{\ell,a}|$$

$H_\ell^{(\text{iv})}$ is symmetric and limited to $[0, 2]$. This measure especially emphasizes large changes of probabilities.

3.2 Different calculations of symbol weights $r_{\ell,a}$

3.2.1 Change of probability (symbol)

The change of probability (symbol) is a measure for the change of symbol-specific probability relative to the sum of absolute symbol-specific probability differences of the given probability distributions. The change of probability (symbol) of symbol a at position ℓ is denoted by $r_{\ell,a}^{(\text{i})}$ and given by

$$r_{\ell,a}^{(\text{i})} = \begin{cases} \frac{p_{\ell,a} - q_{\ell,a}}{\sum_{a' \in \mathcal{A}} |p_{\ell,a'} - q_{\ell,a'}|} & \text{if } p_\ell \neq q_\ell \\ 0 & \text{otherwise.} \end{cases}$$

$r_{\ell,a}^{(\text{i})}$ is antisymmetric and limited to $[-\frac{1}{2}, \frac{1}{2}]$. This measure especially emphasizes a large change of symbol–probability. For each position of the difference logo, the height of the symbol stack with negative measures $r_{\ell,a}^{(\text{i})}$ is equal to the height of the symbol stack with positive measures $r_{\ell,a}^{(\text{i})}$, because each gain of symbol–probability implies a loss of probability for the remaining symbols and vice versa.

3.2.2 Change of information content (symbol)

The change of information content (symbol) is a measure for the symbol-specific change of information content relative to the sum of absolute symbol-specific differences of information content of the given probability distributions. The change of information content

(symbol) of symbol a at position ℓ is denoted by $r^{(\text{ii})}_{\ell,a}$ and given by

$$r^{(\text{ii})}_{\ell,a} = \begin{cases} \frac{p_{\ell,a} H^p_\ell - q_{\ell,a} H^q_\ell}{\sum_{a \in \mathcal{A}} |p_{\ell,a} H^p_\ell - q_{\ell,a} H^q_\ell|} & \text{if } p_\ell \neq q_\ell \\ 0 & \text{otherwise.} \end{cases}$$

$r^{(\text{ii})}_{\ell,a}$ is antisymmetric and limited to $[-1, 1]$. This measure especially emphasizes a large change of symbol-specific information content.

	Change of probability (symbol)	Change of information content (symbol)
Jensen–Shannon divergence	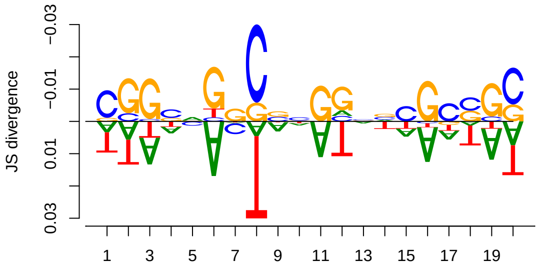	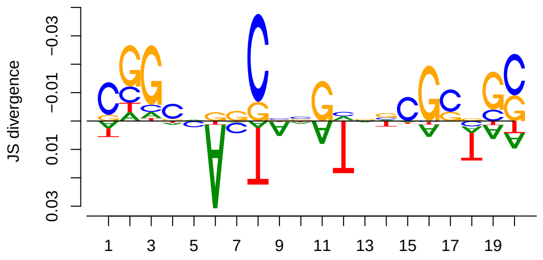
Change of information content (stack)	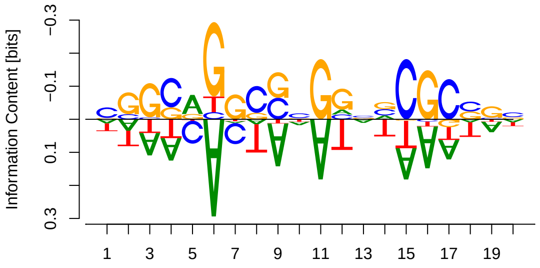	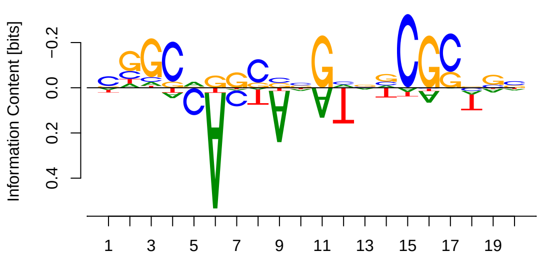
Relative change of information content	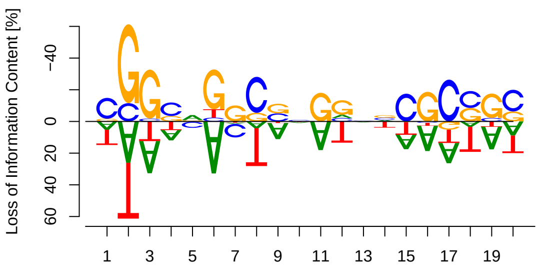	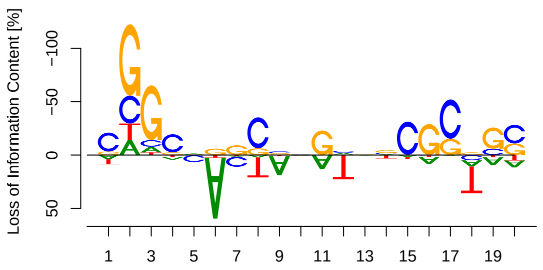
Change of probabilities (stack)	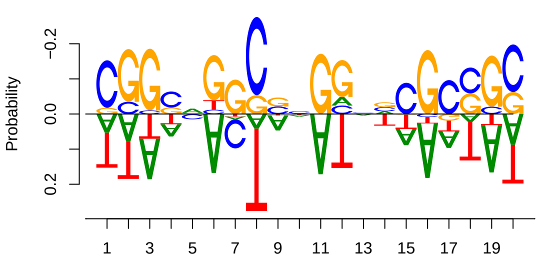	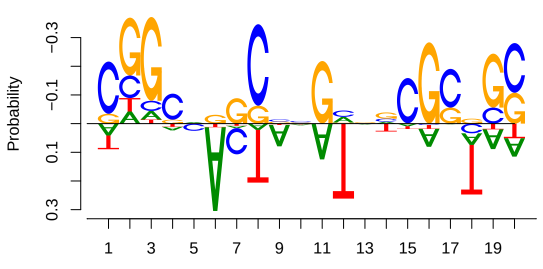

Table S1: **Comparison of different stack heights and symbol weights using two pairs of CTCF motifs.** We compare the four measures 'Jensen–Shannon divergence' (row 1), 'Change of information content (stack)' (row 2), 'Relative change of information content' (row 3), and 'Change of probabilities (stack)' (row 4) for the stack heights and the two measures 'Change of probability (symbol)' (column 1) and 'Change of information content (symbol)' (column 2) for the symbol weights. Depending on the measures used, we emphasize different facets of distribution differences and consequently, the difference logos change dramatically.

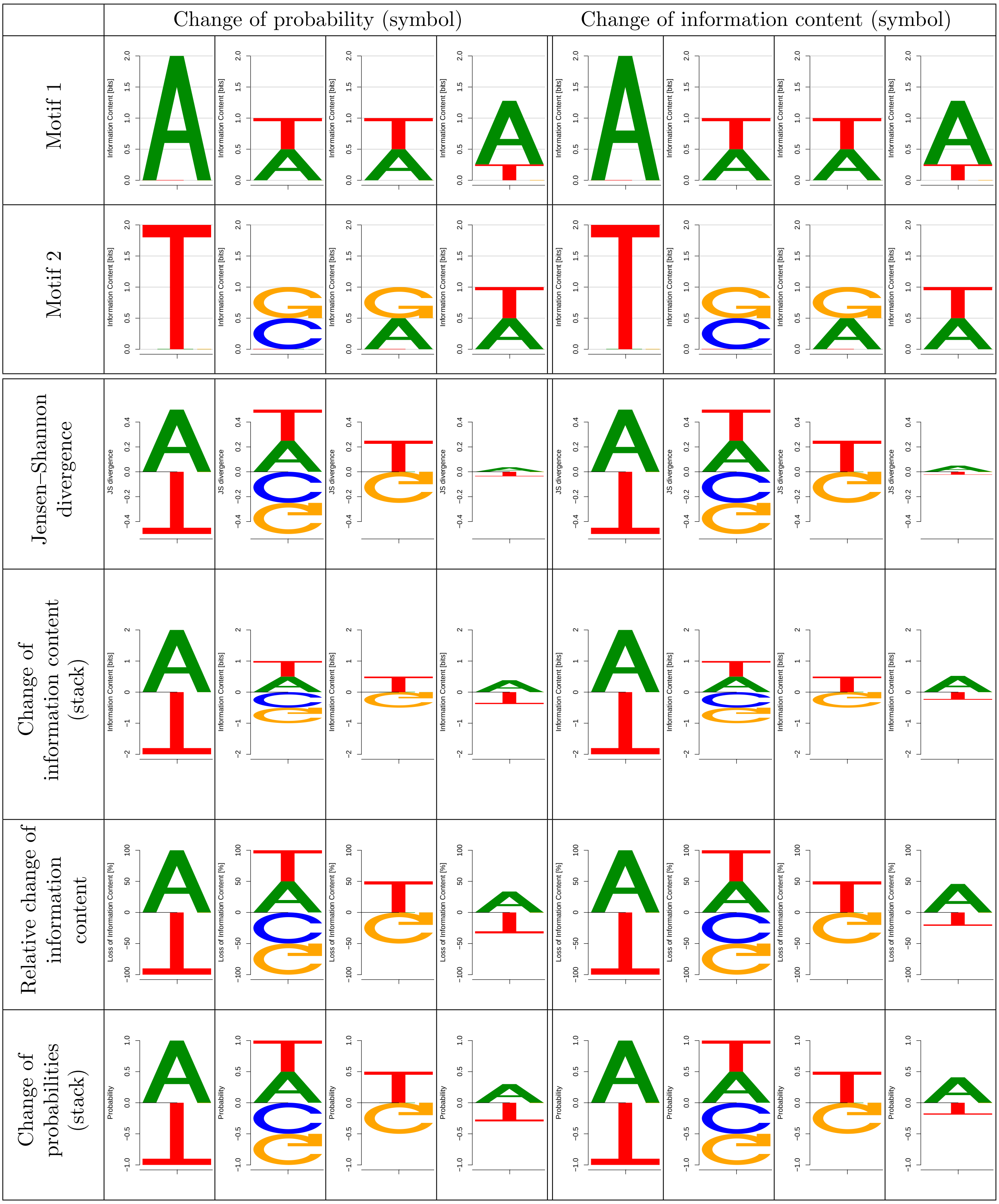

Table S2: **Exemplary comparison of different stack heights and symbol weights using four artificial DNA motifs of length one.**

4 Tool comparison

We compare *DiffLogo*[1] with the five tools *seqLogo*[2], *iceLogo*[3], *MotifStack*[4], *STAMP*[5], and *Two Sample Logo*[6]. From the set of nine CTCF motifs (see 2) we selected the pair of motifs with the highest similarity regarding to the Jensen-Shannon divergence (GM12878 and K562) and the pair of motifs with the lowest similarity regarding to the Jensen-Shannon divergence (H1-hESC and HUVEC) to compare the different tools. We generated a set of 250 alignments for each of the four motifs to guarantee that every tool has the same input.

We generated four sequence logos using the R package *seqLogo* (see Table S3, Row 1). Using the *iceLogo* web page we generated four figures: GM12878 vs K562 and H1-hESC vs HUVEC (see Table S3, Row 2) and K562 vs GM12878 and HUVEC vs H1-hESC (see Table S3, Row 3). It was not possible to install the R package *MotifStack* properly because of multiple errors during installation process ('installation of package 'motifStack' had non-zero exit status'). We generated a stack of sequence logos using the *STAMP* web page (see Figure S4). STAMP does not display a cluster tree, because all branch lengths are equal to zero. Using the *Two Sample Logo* web application, we generated figures for GM12878 vs K562 and H1-hESC vs HUVEC (see Table S3, Row 4). We also used *DiffLogo* to generate the two corresponding difference logos (Table S3, Row 5). For *Two Sample Logo* and *DiffLogo*, the comparison of K562 vs GM12878 and HUVEC vs H1-hESC are symmetric and not shown.

[1] https://github.com/mgledi/DiffLogo
[2] http://www.bioconductor.org/packages/release/bioc/html/seqLogo.html
[3] http://iomics.ugent.be/icelogoserver/
[4] http://www.bioconductor.org/packages/release/bioc/html/motifStack.html
[5] http://www.benoslab.pitt.edu/stamp/
[6] http://www.twosamplelogo.org/

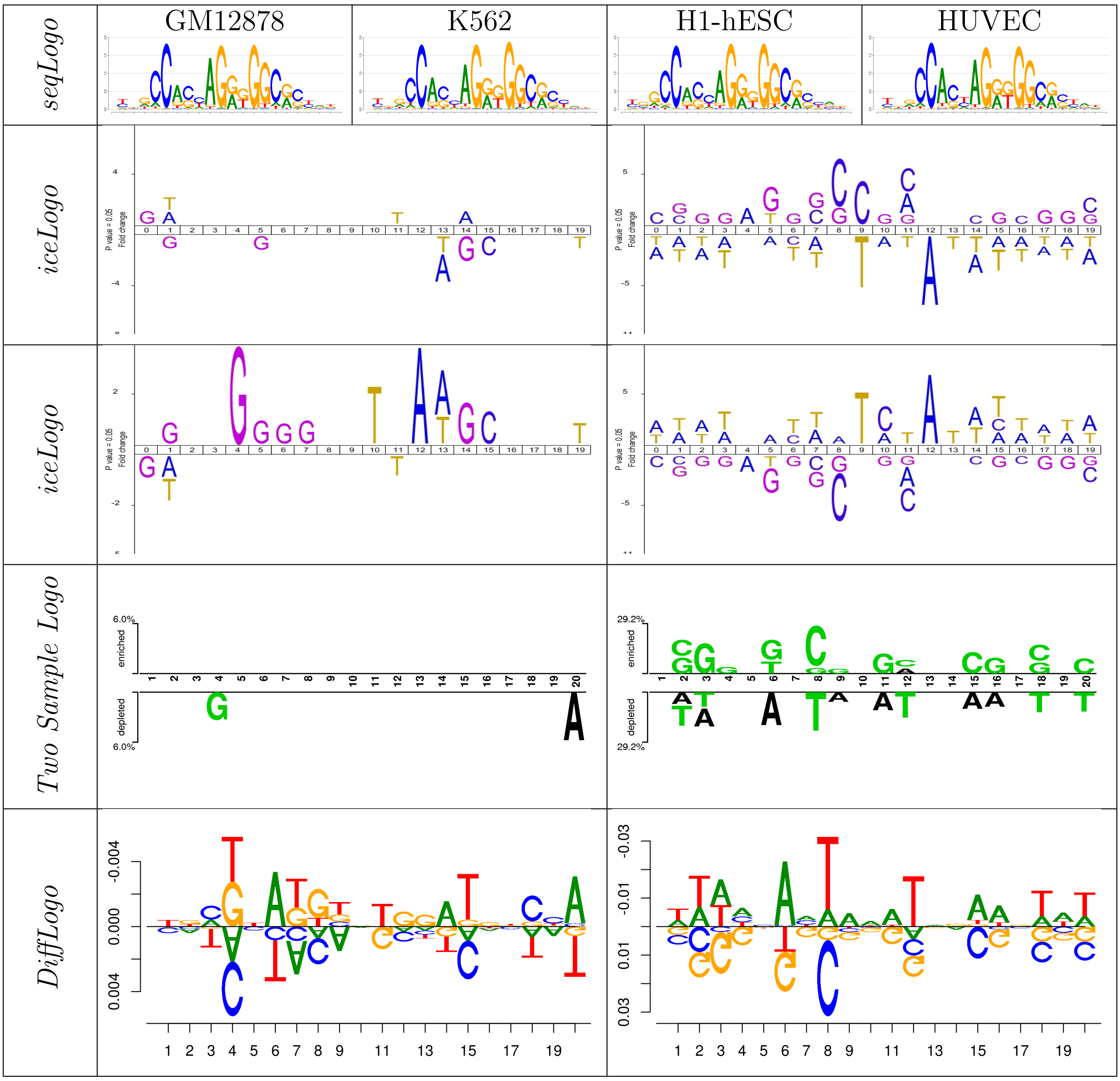

Table S3: Comparison of *seqLogo*, *iceLogo*, *Two Sample Logo*, and *DiffLogo* using two pairs of CTCF motifs.
Row 1: *seqLogo*: Sequence logos of the CTCF motifs for GM12878, K562, H1-hESC, and HUVEC generated by *seqLogo*.
Row 2: *iceLogo*: Logos of the CTCF motifs GM12878 vs K562 and H1-hESC vs HUVEC generated by *iceLogo*.
Row 3: *iceLogo*: Logos of the CTCF motifs K562 vs GM12878 and HUVEC vs H1-hESC generated by *iceLogo*.
Row 4: *Two Sample Logo*: Logos of the CTCF motifs GM12878 vs K562 and H1-hESC vs HUVEC generated by *Two Sample Logo*.
Row 5: *DiffLogo*: Difference Logos of the CTCF motifs GM12878 vs K562 and H1-hESC vs HUVEC generated by *DiffLogo*.

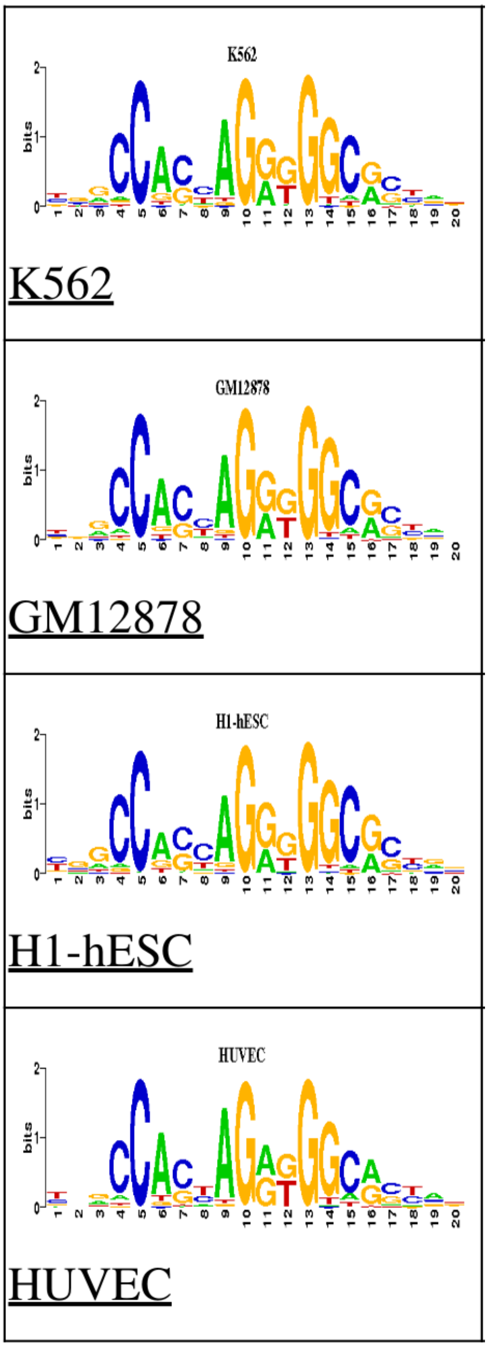

Figure S4: **STAMP.** Stack of sequence logos of the CTCF motifs for GM12878, K562, H1-hESC, and HUVEC generated by STAMP.
